# Occurrence and Toxicity Mechanisms of Perfluorononanoic Acid, Perfluorodecanoic Acid, and Perfluoroundecanoic Acid in Fish: A Review

**DOI:** 10.3390/toxics13060436

**Published:** 2025-05-26

**Authors:** Emma Ivantsova, Amany Sultan, Christopher J. Martyniuk

**Affiliations:** 1 Center for Environmental and Human Toxicology, Department of Physiological Sciences, College of Veterinary Medicine, University of Florida, Gainesville, FL 32611, USA; eivantsova@ufl.edu (E.I.); amanysultan2025@gmail.com (A.S.); 2 Animal Health Research Institute, Agriculture Research Center (ARC), Giza 3751254, Egypt; 3 UF Genetics Institute, Interdisciplinary Program in Biomedical Sciences Neuroscience, University of Florida, Gainesville, FL 32611, USA

**Keywords:** aquatic toxicology, fish, perfluorinated compounds, ecotoxicology

## Abstract

Per- and polyfluoroalkyl substances (PFAS) are used in consumer products and manufacturing. Perfluorononanoic acid (PFNA), perfluorodecanoic acid (PFDA), and perfluoroundecanoic acid (PFUnDA) are long-chain PFAS composed of 9, 10, and 11 carbons, respectively, which exert sublethal toxicity to aquatic species. Here, we review the data regarding the environmental fate and ecotoxicology of these understudied long-chain PFAS in fish. The objectives of this study were to (1) compile the literature to compare physiological or molecular signaling pathways disrupted by PFNA, PFDA, or PFUnDA; and (2) uncover potential biomarkers and pathways of toxicity of longer-chain PFAS using gene ontology computational approaches to shed light on their mechanism of action. Studies show that PFAS have a range of effects on fish, including developmental issues, changes in gene expression, and behavioral modifications. Based on our review, PFNA has been studied more frequently in fish compared to PFDA and PFUnDA; however, longer-chained PFAS are proposed to pose greater toxicity. Based on the computational approach, prominent pathways affected by PFNA include insulin signaling [“Insulin -> CEBPA/CTNNB/FOXA/FOXO”, “Insulin -> STAT Expression Targets”], immune system signaling [“TNF -> STAT Expression Targets”, “IL6 Expression Targets”, and “IL2 Expression Targets”], and growth hormone/prolactin signaling [“GH1/PRLR Expression Targets”, “PRL/GHR -> STAT Expression Targets”, “PRL/PRLR Expression Targets”]. Several transcripts related to cholesterol metabolism were also affected by PFNA. This review summarizes the current knowledge on the distribution, fate, and ecotoxicology of PFNA, PFDA, and PFUnDA in teleost fish, highlighting potential physiological and molecular responses that could aid in assessing long-chain PFAS toxicity in future studies.

## 1. Introduction: Perfluorononanoic Acid (PFNA), Perfluorodecanoic Acid (PFDA), and Perfluoroundecanoic Acid (PFUnDA)

Per- and polyfluoroalkyl substances (PFAS) are used in diverse consumer and industrial products (e.g., detergents, cookware, fabrics, surfactants). These compounds are human-made and were originally introduced by industries around the late 1930s due to their desirable properties, including being both water- and heat-resistant, as well as PFAS making items non-stick [1]. In the early 1970s, aqueous film forming foam (AFFF) was used by the U.S. military, where PFAS are the predominant active component, and AFFF was directly released into the environment as a main source of PFAS [2]. Despite their frequent use, PFAS are a wildlife and public health concern due to their high persistence in environmental matrices, which can lead to consistent, low-level exposures. The concerns about PFAS arise from the diversity of their toxicity mechanisms as they exhibit unique patterns of physiochemical properties [3,4], involve multiple routes of exposure, and are chemically and thermally stable [5]. PFAS also magnify in the aquatic food web and they persistent in the aquatic environment [4].

The two well-studied PFAS over the past several decades have been perfluorooctanesulfonic acid (PFOS) and perfluorooctanoic acid (PFOA), which each contain an 8-carbon hydrophobic backbone. These chemicals have been reported to accumulate in humans and wildlife (i.e., fish) and are widely distributed and persistent in the environment due to their chemical stability. According to the U.S. Department of Health and Human Services National Toxicology Program [6], due to adverse health effects linked to PFOS and PFOA exposure, including hematological and immune-related impacts [7,8], their usage has been phased out over time. PFOS production and usage was phased out in 2022 and PFOA emissions were eliminated in 2015. Both PFOS and PFOA will be considered as Persistent Organic Pollutants (POP) by the Stockholm Convention, which plans to include long-chain PFAS and their salts [9]. Since the phasing out of PFOS and PFOA, PFAS of different carbon lengths and moieties have been used in industrial products as replacements.

**Figure 1:**
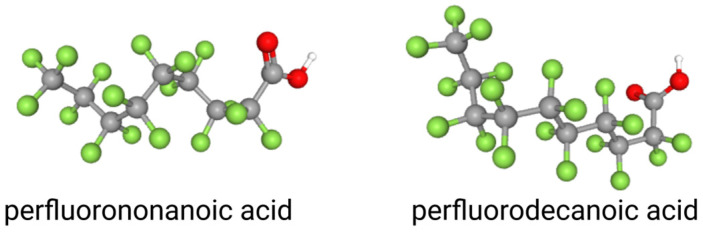
The 3D-structures of perfluorononanoic acid (PFNA) and perfluorodecanoic acid (PFDA). Green depicts the positions of the fluorine, red depicts the position of oxygen, and grey depicts the carbon chain. Images gathered from the National Center for Biotechnology Information (2025). PubChem Compound Summary. Images recovered in January 2025.

Studies generally support the hypothesis that increased chain length contributes to PFAS toxicity [10,11]. While the scientific community has extensive toxicity data for PFOS and PFOA, there is less data available for other closely related PFAS. Perfluorononanoic acid (PFNA), perfluorodecanoic acid (PFDA), and perfluoroundecanoic acid (PFUnDA) are long-chain PFAS, composed of 9, 10, and 11 carbons, respectively, differing from PFOS and PFOA by one to four carbons (Figure 1). In general, PFAS that contain six or fewer carbons in their chain are deemed as short-chain, and those containing seven carbons are deemed as long-chain. Due to their environmental presence and persistence, several long-chain PFAS are being phased out of production [12], yet several studies report the presence of these long-chain PFAS worldwide in the United States of America, Canada, Spain, and Australia [13,14,15,16,17].

According to the Agency for Toxic Substances and Disease Registry (ASTDR) [18], which provides a list containing chemicals that are concerning for their persistence, accumulation, and toxicity to both animals and humans, long-chain PFAS are ranked as followed: PFOS (#147), PFOA (#160), PFDA (#174), PFNA (#194), and PFUnDA (#199). Various PFAS are considered human health concerns as they have been detected in human serum [19,20], urine [21], breast milk [22], and the liver [23]. Elevated human serum concentrations for PFNA, PFDA, and PFUnDA, in addition to both PFOA and PFOS, have been noted globally from Germany [24], France [25], Norway [26], and the USA [20]. In fact, PFNA and PFDA are reported to have half-lives of 3.1 and 7.1 years, respectively, in humans [27]. Long-chain PFAS are also noted as immunotoxicity hazards to human health [28,29], and have been reported to negatively impact cholesterol levels and lipid metabolism [30,31], alter liver and kidney function [31,32], increase the risk of fatty liver disease [33], cardiovascular disease [30], and cause endocrine disruption [34]. In addition to impacting human health, PFAS are known to negatively impact fish health as there is growing evidence that PFAS can interfere with development [35,36,37], decrease survival and growth rates [38,39], induce neurotoxicity [40], endocrine disruption [41,42], oxidative stress and hepatotoxicity [43], immune dysfunction [44], thyroid dysfunction [45,46], and alter lipids [37].

## 2. Objectives and Methodology of the Review

**Figure 2:**
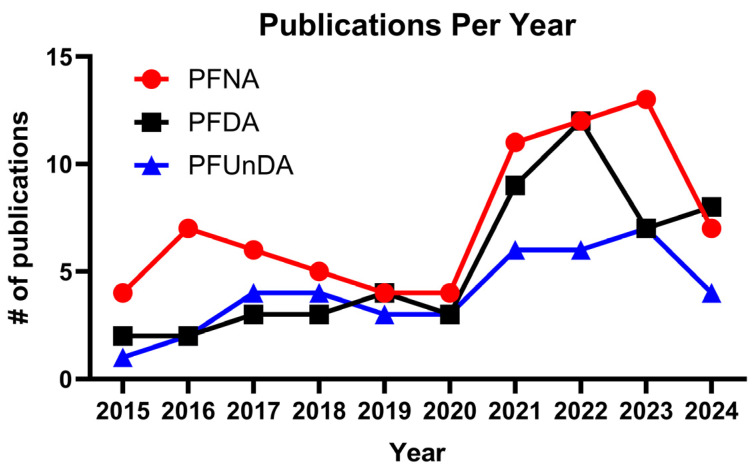
Number of studies conducted in fish for perfluorononanoic acid (PFNA), perfluorodecanoic acid (PFDA), and perfluoroundecanoic acid (PFUnDA). The graph depicts the number of studies on these compounds over the past decade (2014–2024) (key word “the specific PFAS” + fish in NCBI, 23 January 2025).

Toxicity of short-chain PFAS have recently been reviewed in fish species [47,48]. In this review, we compiled data on sub-lethal toxicity for longer-chain PFAS, specifically PFNA, PFDA, and PFUnDA, in fish for improving risk assessment of these chemicals of concern. Google Scholar and NCBI PubMed were used for literature searches [search terms “PFNA” “PFDA” “PFUnDA” + “fish”]. The number of publications retrieved by year is indicated in Figure 2. Studies were included in this review based on the following criteria: reported data on chemical concentration in fish tissue, as well as data describing an exposure (e.g., waterborne or via diet) to these chemicals in any species of fish. This comprehensive review synthesizes knowledge regarding the environmental fate and ecotoxicology of PFNA, PFDA, and PFUnDA in relation to fish species. The objectives of this review were to (1) compile literature to reveal both molecular and physiological endpoints perturbed by these major long-chain PFAS; (2) conduct a computational assessment to reveal sensitive biomarkers of exposure. We also discuss the distribution, accumulation, and sub-lethal toxicity data of these PFAS globally, and provide recommendations for future investigations.

## 3. Environmental Presence

Many studies have reported the presence of PFNA, PFDA, and PFUnDA in various environmental matrices (i.e., water, soil, air) across several continents (Table 1). Concerning water samples, in the United States, PFNA, PFDA, and PFUnDA were found at maximum concentrations of 14 ng/L, 5.8 ng/L, and 1.9 ng/L, respectively, in surface waters from Rhode Island and the New York Metropolitan Area [49]. In the Conasauga River, which runs through Georgia and Tennessee, between 3.74–160 ng/L PFDA and 12.3–456 ng/L PFNA were reported [50]. Various concentrations have also been reported in Florida. In surface water across the state, Camacho et al. [51] reports a mean concentration of 2 ng/L for PFNA and PFDA and 3 ng/L for PFUnDA. Additionally, ranges of 0.1–351.8 ng/L PFNA, 0.4–27.1 ng/L PFDA, and 0.3–114.3 ng/L PFUnDA were documented whereas Griffin et al. [52] reports ranges of 0.21–2.74 ng/L PFNA, 0.24–1.42 ng/L PFDA, and 0.26–0.33 ng/L PFUnDA. Holden et al. [53] evaluated freshwater springs and measured 0.13–0.42 ng/L PFNA and 0.17 ng/L PFDA. Across various Air Force sites in the United States, including Alaska, Anderson et al. [2] evaluated the occurrence of PFNA, PFDA, and PFUnDA in surface water and groundwater and reported 10 μg/L and 3 μg/L PFNA, 3.2 μg/L and 1.8 μg/L PFDA, 0.21 μg/L and 0.086 μg/L PFUnDA, respectively. In the Nordic Region, Sörengård et al. [54] evaluated maximum concentrations of PFNA, PFDA, and PFUnDA in groundwater, river water, and drinking water from a Swedish aquifer. PFNA was found at 11 ng/L, 13 ng/L, and <5 ng/L, respectively, PFDA was found at 22 ng/L, 20 ng/L, and <1.3 ng/L, respectively, and PFUnDA was found at 6.5 ng/L, 1.9 ng/L, and <1.2 ng/L, respectively. Also in Sweden, Koch et al. [55] evaluated freshwater across airports and discovered maximum concentrations of 0.36 ng/L PFNA, 0.16 ng/L PFDA, and 0.03 ng/L PFUnDA. In Norway, seepage water samples contained 26–36 ng/L PFNA, 4.2–5.3 ng/L PFDA, and 8.6–18 ng/L PFUnDA [56], and a mean concentration of 2700 ng/L PFNA was reported in a ditch in Finland [57]. 

In Asia, Naile et al. [58] detected 1.38–14.3 ng/L, 0.23–15.4 ng/L, and up to 3.52 ng/L PFNA, PFDA, and PFUnDA, respectively, in water samples collected from the west coast of Korea. More studies report the occurrence of these compounds in Chinese waters. For instance, PFNA, PFDA, and PFUnDA were found at maximum concentrations of 3.41 ng/L, 2.42 ng/L, and 1.62 ng/L, respectively, in surface waters around three international airports in China [59]. Also in China, seawater samples collected from the Yangtze River Estuary contained 0.03–0.38 ng/L PFNA, 0.09–0.82 ng/L PFDA, and 0.05–0.26 ng/L PFUnDA [60]. Lastly, PFDA and PFNA ranged from 0.13–0.66 ng/L and 0.40–1.43 ng/L, respectively, in the Baiyangdian Lake, China [61]. In Canada, water collected from a creek in Toronto exhibited concentrations (average ± standard deviation) of 2.1 ± 0.2 PFNA and 1.3 ± 0.4 ng/L PFDA [62] and PFNA, PFDA, and PFUnDA ranged from 0.39–0.56 ng/L, 0.060–0.079 ng/L, and up to 0.018 ng/L, respectively, in the St. Lawrence River, Quebec, Canada [13]. Roscales et al. [14] collected water samples from five riverine basins in Spain, including Catalonian, Duero, Eastern Cantabrian, Ebro, and Tagus. Concerning PFNA, measured concentrations ranged from 0.08–0.23 ng/g. Concerning PFDA, it ranged from 0.20–6.7 ng/L. With PFUnDA, concentrations ranged from 0.42–8.8 ng/g. Lastly, Thompson et al. [15] detected median concentrations of 1.2 ng/L PFDA in the Parramatta River, Australia.

Concerning soil and sediment concentrations, Anderson et al. [2] reported 23 μg/kg and 59 μg/kg PFNA, 15 μg/kg and 59 μg/kg PFDA, and 10 μg/kg and 14 μg/kg PFUnDA in surface soil and sediment, respectively, across the United States. In soil and sediment collected from a Swedish aquifer, maximum concentrations of 1.5 ng/g dry weight (dw) and 6 ng/g dw PFNA, 1.2 ng/g dw and 1.3 ng/g dw PFDA, and 0.52 ng/g dw, and 4.5 ng/g dw PFUnDA were reported, respectively [54]. Also in the United States, Griffin et al. [52] reports 0.01–0.82 ng/g PFNA, 0.02–0.26 ng/g PFDA, and 0.02–0.43 ng/g PFUnDA in Florida sediment. In China, 0.02–0.15 ng/g dw PFNA, 0.02–0.11 ng/g dw PFDA, and 0.03–0.13 ng/g dw PFUnDA, were reported from sediment collected from the Yangtze River Estuary, China [60], and Ibor et al. [63] detected 1.0, 5.0, and 0.6 ng/g of PFNA, PFDA, and PFUnDA, respectively, in soil from a solid waste dumpsite in Calabar, Nigeria. In the St. Lawrence River, Québec, Canada, PFNA, PFDA, and PFUnDA were found at concentrations of up to 0.042 ng/g dw, 0.031 ng/g dw, and 0.071 ng/g dw, respectively [13], and 112 pg/g dw PFNA, 298 pg/g dw PFDA, and 138 pg/g dw PFUnDA were detected in sediment collected from Port of Santos, Brazil [64].

**Table 1:** Environmental presence of perfluorononanoic acid (PFNA), perfluorodecanoic acid (PFDA), and perfluoroundecanoic acid (PFUnDA) across multiple matrices.

Location	Matrices	PFNA	PFDA	PFUnDA	Cite
Air Force sites across the U.S.	Surface soil	23 ng/g	15 ng/g	10 ng/g	[2]
	Sediment	59 ng/g	59 ng/g	14 ng/g	[2]
	Surface water	10,000 ng/L	3200 ng/L	210 ng/L	[2]
	Groundwater	3000 ng/L	1800 ng/L	86 ng/L	[2]
Florida	Freshwater	0.13–0.42 ng/L	0.17 ng/L		[53]
	Surface water	0.1–351.8 ng/L	0.4–27.1 ng/L	0.3–114.3 ng/L	[51]
	Surface water	0.21–2.74 ng/L	0.24–1.42 ng/L	0.26–0.33 ng/L	[52]
	Sediment	0.01–0.82 ng/g	0.02–0.26 ng/g	0.02–0.43 ng/g	[52]
Georgia and Tennessee	Surface water	12.3–456 ng/L	3.74–160 ng/L		[50]
Rhode Island and the New York Metropolitan Area	Surface water	14 ng/L	5.8 ng/L	1.9 ng/L	[49]
Sweden	Groundwater	11 ng/L	22 ng/L	6.5 ng/L	[54]
	River water	13 ng/L	20 ng/L	1.9 ng/L	[54]
	Drinking water	<5 ng/L	<1.3 ng/L	<1.2 ng/L	[54]
	Freshwater	0.36 ng/L	0.16 ng/L	0.03 ng/L	[55]
	Soil	1.5 ng/g dw	1.2 ng/g dw	0.52 ng/g dw	[54]
	Sediment	6 ng/g dw	1.3 ng/g dw	4.5 ng/g dw	[54]
Finland	Freshwater	2700 ng/L			[57]
Norway	Seepage water	26–36 ng/L	4.2–5.3 ng/L	8.6–18 ng/L	[56]
Korea	Brackish water	1.38–14.3 ng/L	0.23–15.4 ng/L	3.52 ng/L	[58]
China	Seawater	0.03–0.38 ng/L	0.09–0.82 ng/L	0.05–0.26 ng/L	[60]
	Freshwater	0.40–1.43 ng/L	0.13–0.66 ng/L		[61]
	Sediment	0.02–0.15 ng/g dw	0.02–0.11 ng/g dw	0.03–0.13 ng/g dw	[60]
Spain	Freshwater	0.08–0.23 ng/g	0.20–6.7 ng/L	0.42–8.8 ng/g	[14]
Australia	Freshwater		1.2 ng/L		[15]
Canada	Freshwater	2.1 ± 0.2 ng/L	1.3 ± 0.4 ng/L		[62]
	Freshwater	0.39–0.56 ng/L	0.060–0.079 ng/L	0.018 ng/L	[13]
	Sediment	0.042 ng/g dw	0.031 ng/g dw	0.071 ng/g dw	[13]
Brazil	Sediment	112 pg/g dw	298 pg/g dw	138 pg/g dw	[64]
Nigeria	Soil	1.0 ng/g	5.0 ng/g	0.6 ng/g	[63]

## 4. Concentrations of PFNA, PFDA, and PFUnDA in Fish Tissues

**Figure 3:**
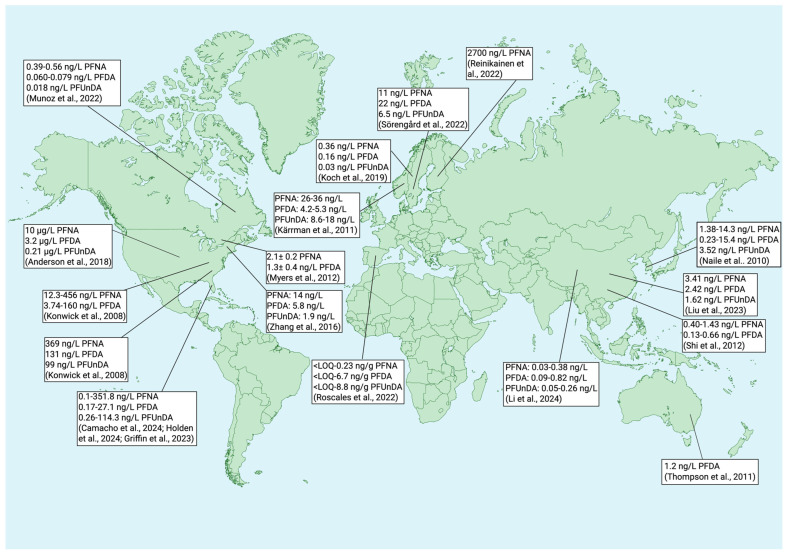
Concentrations of perfluorononanoic acid (PFNA), perfluorodecanoic acid (PFDA), and perfluoroundecanoic acid (PFUnDA) in water around the globe. Created with BioRender.com. ([2] Anderson et al., 2018; [13] Munoz et al., 2022; [14] Roscales et al., 2022; [15] Thompson et al., 2011; [49] Zhang et al., 2016; [50] Konwick et al., 2008; [51] Camacho et al., 2024; [52] Griffin et al., 2023; [53] Holden et al., 2024; [54] Sörengård et al., 2022; [55] Koch et al., 2019; [56] Kärrman et al., 2011; [57] Reinikainen et al., 2022; [58] Naile et al., 2010; [59] Liu et al., 2023; [60] Li et al., 2024; [61] Shi et al., 2012; [62] Myers et al., 2012).

PFNA, PFDA, and PFUnDA have been detected in tissues in various species and in different environmental matrices (Figure 3). Labadie and Chevreuil [65] detected all four of these PFAS in European chub tissue, in which they ranged from 43.1–4997.2 ng/g. Plasma was found to have the highest PFAS concentration, followed by liver, gills, gonads, and muscles. Fair et al. [66] analyzed the presence of different PFAS, including PFDA, PFNA, and PFUnDA, in whole and muscle fillets samples of Atlantic croaker (*Micropogonias undulatus*); red drum (*Sciaenops ocellatus*); spot (*Leiostomus xanthurus*), spotted seatrout (*Cynoscion nebulosus*), and striped mullet (*Mugil cephalus*). At least 30% of the samples tested had detectable levels of PFDA, PFNA, and PFUnDA. Concerning whole samples, the average relative percentage of PFNA, PFDA, and PFUnDA, presented as the percent of total PFAS in ng/g wet weight (ww), ranged from 2.49–3.56, 9.3–15, and 4.97–21.7, respectively, and, in fillets, ranged from 2.6–9.47, 9.04–17.7, 1.39–8.53. In another study, Fujii et al. [67] found that 33% of perfluoroalkyl carboxylic acid (PFCA) compounds detected in the USA and Canada and 96% of PFCAs in Japan and Korea were long chained, which included our four compounds of focus. Edible muscle from Pacific cod in both Korean and Japanese coastal waters had up to 892 pg/g ww and 1710 pg/g ww, respectively, while 216–670 pg/g ww was detected in the muscle of pacific cod from the northeast Pacific Ocean. In another study, Munoz et al. [13] analyzed the range of PFAS in bluntnose minnow (*Pimephales notatus*), emerald shiner (*Notropis atherinoides*), gold shiner (*Notemigonus crysoleucas*), northern pike (*Esox lucius*), pumpkinseed (*Lepomis gibbosus*), rock bass (*Ambloplites rupestris*), sand shiner/mimic shiner (*Notropis stramineus*/*Notropis volucellus*), sicklefin redhorse (*Moxostoma* spp.), smallmouth bass (*Micropterus dolomieu*), white sucker (*Catostomus commersonii*), and yellow perch (*Perca flavescens*) from the St. Lawrence River, Quebec, Canada. PFDA and PFUnDA had a detection frequency of 100% in the fish tested and ranged from 0.62–7.3 ng/g ww and 0.45–6.8 ng/g ww, respectively. PFNA was detected by 94.7% and was found up to 8.2 ng/g ww in fish. Most recently, Barbo et al. [68] analyzed the presence of PFAS in 501 samples of fish fillets collected from 2013 to 2015 in the United States. Of the 13 PFAS analyzed, PFOS was the major compound detected, with a total percentage contribution of 74.2%, followed by PFUnDA (9.6%), PFDA (6.7%), and PFNA (2.8%). Noteworthy, Kirkeli et al. [69] detected PFDA, PFNA, and PFUnDA in fish liver from downstream of Jebel Aulia dam at Nile river, Sudan, at concentrations of 137 ± 12, 143 ± 4, and 4 μg/kg ww, respectively, while Melake, Bervoets [70] observed accumulation of PFNA, PFDA, and PFUnDA in fish tissues from Lake Hawassa, Ethiopia. Muscles of Nile tilapia (*Oreochromis niloticus*), sharptooth catfish (*Clarias gariepinus*), and East African ray-fined fish (*Barbus intermedius*) contained 0.25, 0.188, and 0.26 ng/g ww PFDA, respectively, and 0.226, 0.18, and 0.92 ng/g ww PFUnDA, respectively. PFNA was only detected at 0.62 ng/g ww in *O. niloticus*. In liver tissue, 1.43 and 0.26 ng/g ww PFDA and 0.93 and 0.23 ng/g ww PFUnDA were reported in *C. gariepinus* and *B. intermedius*, respectively. PFNA was only detected in *C. gariepinus* at 0.2 ng/g ww. The study concluded that consumption of those PFAS-contaminated fish had no risk to human health.

## 5. Metabolism and Excretion

Currently, there is no indication of a significant decline in environmental levels of PFAS [71]. PFAS are known as “forever chemicals” as they are environmentally stable and breakdown very slowly due to their highly stable perfluorocarbon moieties [72]. Once in the environment, PFAS infiltrate, bioaccumulate, and biomagnify in biota, which can lead to trophic transfer [55,73,74,75]. The strong covalent bonds between carbon and fluorine found within PFAS are the primary contributing factor towards their degradation resistance and concentration magnification in the environment [76].

Environmental accumulation of PFAS is dependent on their chemical properties and on environmental conditions as PFAS bind to particulate organic matter by electrostatic interactions [52]. For example, long-chain PFAS mostly accumulate in the sediment due to their high sorption affinity and hydrophobicity. Tang et al. [77] reported high concentrations of PFUnDA (0.002–0.687 ng/g dw) in sediment samples collected from three major rivers in Hainan Island, China and determined this was due to the chemical’s high fluorocarbon content, low solubility (0.001 mg/L), and its high hydrophobicity (Log Kow = 6.8). On the other hand, Hu et al. [78] established a molecular dynamic model and density functional theory to study the environmental fate of PFAS in which molecular dynamics showed that medium- or long-chain PFAS aggregate spontaneously to submicelles in the aquatic environments; thus, promoting their bioaccumulation. The aggregates can encapsulate PFAS molecules, which prevents their degradation, and the van der Waals interactions, not the electrostatic effects, were found to cause the aggregated PFAS to dissolve into the lipid membrane matrix. These results are supported by an additional molecular dynamic study that showed that the key pathway of PFAS bioaccumulation is their partitioning into the membrane phospholipids [79].

Overall, it has been determined that long-chain PFAS possess higher bioaccumulation potential in fish and the environment compared to short-chain PFAS due to their chemical properties [80]. Though long-chain PFAS are known for their greater bioaccumulation, short-chain PFAS are more mobile and are challenging to remove from water by treatments [81]. Based on recent studies, it has been implied that the hydrophobic carbon–fluorine chains of PFAS occupy binding sites of specific target protein, and hydrogen bonds are formed between the amino acid residues and the acid group from the PFAS [82]. Noteworthy is that the protein binding affinities of PFAS alternatives change according to their structural formulas. Additionally, transport proteins play a pivotal role in PFAS distribution and accumulation inside the body as PFAS could alter the structure of the binding proteins and might impact their transport function. Thus, long-chain PFAS can trigger molecular events through their binding to specific molecular targets in different organisms [83], leading to their stability and accumulation in aquatic species. Some of these molecular targets are revealed in Section 6.5. While data remain scarce for degradation and metabolism, the Office of Environmental Health Hazard Assessment [27] reports that long-chain PFAS, like PFNA and PFDA, are not significantly metabolized in animals and humans, leading to their accumulation and increasing toxicity potential.

## 6. Exposures and Adverse Effects

### 6.1. Developmental Effects

Like PFOA and PFOS, adverse outcomes of other long-chain PFAS have been examined in developing fish embryos and larvae (Table 2). Concerning PFNA, following exposure to 9281.6 ng/L, 92,816 ng/L, or 0.928 mg/L until 5 dpf, Jantzen et al. [35] found that embryonic zebrafish (*Danio rerio*) did not show any signs of delayed development, embryonic abnormalities, mortality, or changes in interocular distance. However, 0.928 mg/L PFNA was found to significantly reduce body length and increase yolk sac size. Control fish had an average body length of 4.72 ± 0.24 mm whereas the exposed fish had an average body length of 4.63 ± 0.11 mm. Regarding yolk sac size, control fish had an average area of 0.43 ± 0.04 mm^2^ and exposed fish had an average area of 0.45 ± 0.05 mm^2^. Liu et al. [84] exposed zebrafish embryos to up to 185.6 mg/L PFNA for 96 h. PFNA was found to be acutely toxic where the half-lethal concentration at 8 and 24 hpf were 158.7 and 140.1 mg/L, respectively. The rate of gastrulation increased at concentrations above 139.2 mg/L, hatching rate decreased in a dose-dependent manner, and the rate of ventricular edema increased in a dose-dependent manner. In a more recent study, Rericha et al. [85] analyzed mortality and conducted an embryonic photomotor response at 24 hpf and larval photomotor response at 120 hpf in zebrafish exposed to up to 100 µM of 58 different PFAS, including PFDA and PFNA. PFNA was one of the 58 compounds that elicited morphological effects or mortality, and the lowest concentration for these effects was 34.7 mg/L.

Less toxicity data is available for PFDA and PFUnDA relative to PFNA. Truong et al. [36] completed a developmental toxicity screen on dechorionated zebrafish embryos following exposure to 139 PFAS from 6 to 120 hpf at varying concentrations (0.015–100 µM). Eight PFAS commonly studied in vivo were ranked in potency (from their morphological effects), in which PFDA was found to be the most potent teratogen as it had the lowest benchmark dose of 0.114 mg/L. Zebrafish embryos were also exposed to 0.5, 1, and 2 mg/L PFDA until 72 hpf by Xiao et al. [86] in which embryo length was significantly shorter with treatment groups and mortality decreased in a dose-dependent manner. Regarding PFUnDA, Kim et al. [41] exposed zebrafish embryo/larvae for 5 days to 0.03, 0.1, or 0.3 mg/L PFUnDA and found that hatch rate, hatch percentage, and survival were not significantly impacted following exposure; however, swim bladder deflation, yolk sac edema, and heart and jaw malformations were observed in larvae. Specifically, 0.03 and 0.3 mg/L increased yolk sac size and 0.3 mg/L decreased the eyeball size in larvae. Overall, PFNA has been studied more often and investigated at higher concentrations compared to PFDA and PFUnDA in fish. Both PFNA and PFUnDA are reported to induce yolk sac edema and PFDA and PFNA have been shown to negatively affect development and cause high mortality in a dose-dependent manner in fish. However, more data surrounding PFUnDA exposure are needed.

### 6.2. Oxidative Stress

The generation of reactive oxygen species (ROS) is a common response in organisms to chemical exposures, including fish. Multiple studies report oxidative stress in fish exposed to PFNA. Yang et al. [87] exposed zebrafish larvae to 0.2, 0.5, 1, 5, or 15 mg/L PFNA for 140 h. Superoxide dismutase (SOD) was significantly increased in fish treated with 1 and 5 mg/L where the activity of the enzyme increased by 67% and 83%, respectively. Additionally, both SOD and catalase (CAT) activity were decreased and malondialdehyde (MDA) content increased by 100.9% with the highest chemical exposure. Rainieri et al. [88] exposed zebrafish embryos up to 500 mg/L PFNA and found that fish exposed to 10 mg/L resulted in an increase in thiobarbituric acid reactive species (TBARS) concentration, which indicates oxidative damage. Additionally, exposure to 1, 5, 10, or 100 mg/L PFNA caused glutathione S-transferase pi 1 (gstp1) and heat shock protein 70 (hsp70) to be induced at all tested concentrations. Tang et al. [10] exposed embryonic zebrafish to 0.0464, 0.464, or 4.64 mg/L of PFNA for 5 days. ROS was significantly induced in all tested concentrations; MDA was significantly induced at 0.0464 and 4.64 mg/L PFNA, and nitric oxide (NO) and inducible nitric oxide synthase (iNOS) were induced at 4.64 mg/L PFNA. All concentrations inhibited glutathione (GSH). Regarding enzymatic levels, 0.464 and 4.64 mg/L PFNA inhibited glutathione peroxidase (Gpx), 0.0464 and 4.64 mg/L PFNA inhibited SOD, and 4.64 mg/L PFNA inhibited CAT. Additionally, Liu et al. [84] exposed zebrafish embryos to up to 185.6 mg/L PFNA for 96 h and all treatment groups led to a significant ROS production in larvae. Thus, data point to oxidative stress as a mechanism for PFNA-induced toxicity.

Regarding PFDA, Xiao et al. [86] observed a concentration-dependent increase of oxidative stress in zebrafish exposed to 0.5, 1, and 2 mg/L PFDA until 72 hpf. High ROS, reduced CAT and SOD activities, and reduced number of apoptotic cells were observed. Additionally, gene expression of both *p53* and *caspase3* were downregulated, while *bcl-2* expression was upregulated. The study suggested that PFDA can induce immune response by increasing oxidative stress as the number of neutrophils was rescued in fish treated with the highest tested concentration when using the antioxidant astaxanthin. To date, no studies have measured the relationship between oxidative stress and PFUnDA exposure to fish.

### 6.3. Endocrine System

There is some evidence that PFAS can act as endocrine disrupting chemicals [47,48] and most studies have focused on hormonal systems and reproduction. Concerning PFNA, Zhang et al. [89] exposed adult zebrafish to 0.01, 0.1, or 1 mg/L PFNA for 180 days to analyze reproduction-related effects. In the gonads, PFNA concentration increased in a dose-dependent manner. Males were observed to have a higher PFNA concentration in the spermary than in the ovary of females following exposure to 0.01 and 1.0 mg/L. The gonadosomatic index, the weight of the gonad relative to body weight, in males was significantly decreased in all treatments. Additionally, egg production was reduced following exposure to 0.1 mg/L, and a decreased hatching rate was observed following 72 h of exposure to 0.01 and 1.0 mg/L. In both genders, testosterone increased following exposure to 0.01 mg/L and 17β-estradiol was increased following exposure to 0.1 and 1.0 mg/L. In addition, vitellogenin, the egg yolk precursor protein, was significantly increased in male liver in all treatment groups. Jo et al. [90] exposed zebrafish to 0.01, 0.1, 1, or 10 mg/L PFDA for 120 days to assess the potential for long-term endocrine disruption effects. Larval, juvenile, and adult fish survival decreased in a dose-dependent manner. In male fish, 17β-estradiol (E2)/testosterone (T) (E2/T) ratio and E2/11-ketotestosterone (E2/11-KT) ratio were increased in fish exposed to 1 mg/L. Taken together, this evidence suggests that these long-chain PFAS perturb sex steroids and the reproductive axis in fish.

Thyroid hormone signaling alterations have also been notably altered by PFNA [91] and PFUnDA [41]. Liu et al. [91] exposed 23-day old zebrafish to 0.05, 0.1, 0.5, or 1 mg/L PFNA for 180 days. Offspring embryos (F_1_) were also reared from the parental zebrafish (F_0_) and were also exposed for 180 days. In the study, hyperplasia of thyroid follicle cells and hypertrophy of follicular epithelium were found in F_0_ fish exposed to 0.1 and 0.5 mg/L. Additionally, in adult males, 0.05, 0.1, and 1 mg/L increased triiodothyronine (T_3_) levels. In male and female F_1_ adults, T_3_ levels were also elevated in all treatment groups. Taken together, these long-chain PFAS affect different hormone systems; however, other hormone systems have yet to be studied intensely for these specific chemicals in fish, which represents a limitation in our current knowledge.

### 6.4. Behavioral Effects

Behavioral alterations can occur with exposure to environmental pollutants, including perfluorinated substances. Jantzen et al. [35] found that embryonic zebrafish exposure to 9281.6 ng/L, 92,816 ng/L, or 0.928 mg/L PFNA presented with decreased swimming velocity following a 5-day exposure. Additionally, 92,816 ng/L PFNA significantly increased the distance travelled and 92,816 ng/L and 0.928 mg/L caused fish to spend more time in the middle of their tank, indicating stress or anxiety. Menger et al. [92] exposed zebrafish embryos to various PFAS for 6 days to observe behavioral alterations. Following exposure to concentrations ranging 92.8 ng/L to 185.6 mg/L PFNA, 49 µM PFNA was found to significantly increase peak activity and decrease swimming distance during the light period. At 6.96 mg/L and 22.73 mg/L µM, PFNA was also noted to increase the burst activity of larvae in the dark period. Additionally, Rericha et al. [85] conducted an embryonic photomotor response test at 24 hpf and larval photomotor response test at 120 hpf in zebrafish following exposures up to 100 µM of 58 different PFAS, including PFDA and PFNA. No compound tested produced abnormal results during the embryonic photomotor response. During the dark phase, hypoactivity was observed in fish exposed to 16.24 mg/L PFNA where hypoactivity was observed in fish exposed to 51.4 mg/L PFDA and hyperactivity at 1.28 mg/L and 17.99 mg/L PFDA. In the light phase, 1.16 and 16.24 mg/L PFNA induced hypoactivity and 0.464 mg/L and 34.66 mg/L PFNA induced hyperactivity. PFDA only induced hypoactivity and hyperactivity at 8.43 and 1.28 mg/L, respectively. Additionally, Xiao et al. [86] observed a decrease in locomotor activity in zebrafish treated with 0.5, 1, and 2 mg/L PFDA based upon average speed, movement time, and mobility. Taken together, between 0.0928 to 46.4 mg/L PFNA and 0.102 to 51.4 mg/L PFDA have been noted to cause behavioral alterations. Very few studies in literature evaluate the impact PFUnDA on behavior in fish. In a study completed by Rericha et al. [85], PFUnDA did not elicit any behavioral alterations in fish exposed to a single concentration of 0.248 mg/L up to 120 hpf.

**Table 2:** Studies reporting on the ecotoxicology of perfluorononanoic acid (PFNA), perfluorodecanoic acid (PFDA), and perfluoroundecanoic acid (PFUnDA) in fish.

Species	Sex	Dose	Duration	Outcome of Exposure	Reference
Embryonic Zebrafish (*Danio rerio*)	N/A	9281.6 ng/L, 92,816 ng/L, 0.928 mg/L PFNA	5 dpf	No delayed development, embryonic abnormalities or high mortality. Reduced body length and increased yolk sac size. Decreased swimming velocity in all treatments.	[35]
Larval Zebrafish (*Danio rerio*)	N/A	0.2, 0.5, 1, 5, 15 mg/L PFNA	140 h	SOD increased with 1 and 5 mg/L. SOD and CAT activity decreased and MDA content increased with 15 mg/L PFNA.	[87]
Embryonic Zebrafish (*Danio rerio*)	N/A	0–500 mg/L PFNA	N/A	LC50 was 108.6 mg/L. 10 mg/L increased TBARS concentration. Exposure to 1, 5, 10, or 100 mg/L induced *gstp1* and *hsp70*.	[88]
Embryonic and larval Zebrafish (*Danio rerio*)	N/A	0–51.4 mg/L PFDA or 0–46.4 mg/L PFNA	120 hpf	Morphological effects/mortality were observed starting at 74.8 µM. Hypoactivity and hyperactivity were observed in the dark and light phases.	[85]
Embryonic and larval Zebrafish (*Danio rerio*)	N/A	92.8 ng/L–185.6 mg/L PFNA	6 days	49 µM PFNA increased peak activity and decreased swimming distance during the light period of the behavioral assay. 15 µM and 49 µM increased burst activity of larvae in the dark period.	[92]
Adult Zebrafish (*Danio rerio*)	Male and female	0.01, 0.1, 1 mg/L PFNA	180 days	PFNA concentration increased in a dose-dependent manner in gonads. Decreased hatching rate after 3 days of exposure to 0.01 and 1.0 mg/L. Gonadosomatic index in males decreased in all treatments. *Erα*, *erβ*, *Fshr*, *3β-hsd*, and *LHβ* were altered in both sexes.	[89]
Zebrafish (*Danio rerio*)	Male and female	0.01, 0.1, 1, 10 mg/L PFDA	120 days	Survival decreased in a dose-dependent manner. E2/T and E2/11-KT ratios increased with 1 mg/L in males. 1 mg/L increased the expression of *cyp19b*, *erα*, and *er2β*.	[90]
23 dpf Zebrafish (*Danio rerio*)	Male and female	0.05, 0.1, 0.5, or 1 mg/L PFNA	180 days (F_0_) 180 days (F_1_)	Hyperplasia of thyroid follicle cells and hypertrophy of follicular epithelium in 0.1 and 0.5 mg/L F_0_ fish. In F_1_ adults, T_3_ levels were elevated, *TRα* and *TTR* were induced and *Ugt2A1*, *TRβ*, and *TPO* were upregulated.	[91]
Zebrafish (*Danio rerio*)	N/A	0–185.6 mg/L PFNA	96 h	PFNA was acutely toxic. Rate of gastrulation increased above 300 µmol/L. Hatching rate decreased in a dose-dependent manner. All treatments increased ROS. *ucp2* and *lfabp* were upregulated in a dose-dependent manner and *mt-nd1* and *sod1* were downregulated in all treatments.	[84]
Embryonic Zebrafish (*Danio rerio*)	N/A	7711.2 ng/L–51.408 mg/L PFDA and 8461.4 ng/L–56.409 mg/L PFUnDA	6 to 120 hpf	PFDA was the most potent teratogen with a benchmark dose of 0.223 µM. PFUnDA was the 3rd least potent compound ranked.	[36]
Embryonic Zebrafish (*Danio rerio*)	N/A	0.0464, 0.46408, 4.6408 mg/L PFNA	5 days	ROS induced in all concentrations. MDA induced at 0.1 and 10 µg/L and NO and iNOS induced at 10 µg/L. All concentrations inhibited GSH, 1 and 10 µg/L inhibited Gpx, 0.1 and 10 µg/L inhibited SOD, and 10 µg/L inhibited CAT.	[10]
Embryonic Zebrafish (*Danio rerio*)	N/A	92,816 ng/L, 0.928 mg/L, 9.28 mg/L, 92.8 mg/L PFNA	120 hpf	Malformation observed at 200 µM and mortality observed at 20 and 200 µM. Enriched pathways were p53, PPAR, phagosome related. *Amhc*, *nppa*, *nkx2.5*, *end1*, and *tgfb2* were upregulated.	[93]
Rainbow trout (*Oncorhynchus mykiss*)	N/A	200 ppm PFDA (5 mg/kg/day) or 1000 ppm PFNA (25 mg/kg/day)	6 months	Increased liver tumor incidence observed with 200 ppm PFDA. Tumor multiplicity and size increased with PFDA and PFNA treatments. Transcripts represented processes related to the adaptive immune response, apoptosis, cell proliferation, protein translation, and transcription.	[94]
Zebrafish (*Danio rerio*)	N/A	0.03, 0.1, 0.3 mg/L PFUnDA	5 days	*Ugt1ab* upregulated and *trα* and *trβ* showed downregulated trends; however, the trend for *trβ* was stronger.	[41]
Zebrafish (*Danio rerio*)	N/A	0.2, 1, 2 mg/L PFDA	6 days	ROS accumulation and increased CAT and SOD levels. Concentration-dependent behavioral changes related to average speed and mobility.	[86]

### 6.5. Molecular and Cellular Targets

Physiological responses to PFAS can be better understood through transcriptomic responses. Of the compounds studied, expression disruptions due to PFNA have been most studied, where transcriptional pathways related to apoptosis, cardiac dysfunction, lipid metabolism, and oxidative stress have been noted. For example, PFNA-exposed zebrafish showed altered gene expression of transcripts related to free cholesterol levels, as well as apoptosis primarily in the heart, abdomen, and tail. The inhibition of erythropoiesis and cardiotoxic effect of PFNA were also noted and accompanied by decreased heart rate and low blood flow in PFNA exposed fish [95]. A summary of some transcripts regulated by PFNA, PFDA, and/or PFuNDA is presented in Table 3, supporting impacts on biological processes such as endocrine system, oxidative stress, immune function, and lipids.

**Figure 4:**
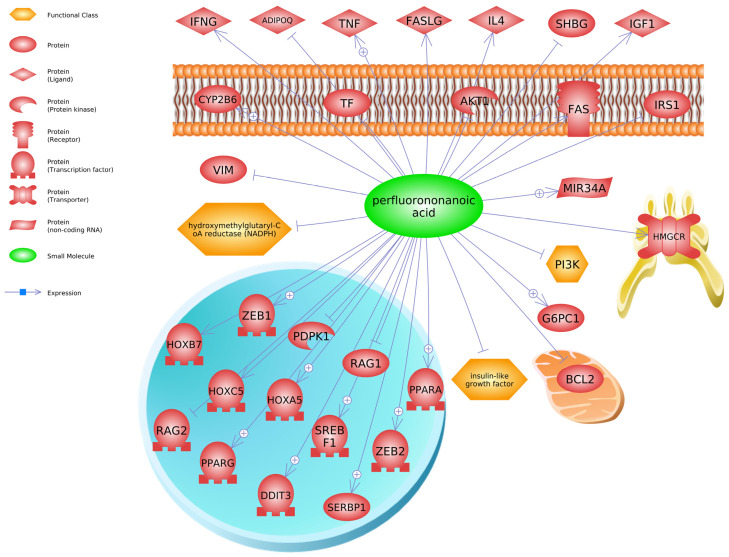
Proteins associated with perfluorononanoic acid (PFNA) exposure (Pathway Studio v12, Elsevier). Abbreviations for genes are provided in Supplementary Material.

To identify additional molecular biomarkers for these long-chain PFAS and gain mechanistic insight into their biological effects, we generated molecular pathways based on transcripts listed in the Comparative Toxicogenomics Database (CTD) [96] for iPathway (see below) or chemical–transcript relationships found in Pathway Studio. We used the gene data to conduct an enrichment analysis in Pathway Studio (Elsevier, version 12), focusing on PFNA as that chemical contained the most gene expression data in the database at the time of the queries. Our computational analysis using Pathway Studio has been described previously by us [48,97]. Briefly, gene set enrichment analysis creates a central “seed” from all relevant entities in the database, to find common effectors (expression targets, binding partners, and post-translational targets). A Kolmogorov–Smirnov test with 1000 permutations was conducted to determine whether specific gene sets were preferentially enriched in the data compared to the background reference probability distribution (this is a database housed in Pathway Studio that has over 1000 gene networks related to cell signaling pathways and processes). If the number of genes in the input list are associated with a cell signaling pathway or processes more so than expected by chance, then the gene set is identified as “enriched”. The enrichment *p*-value for gene seeds was set at *p* < 0.05. Transcripts regulated by PFNA in the database include SREBF1 (sterol regulatory element binding transcription factor 1), PPARA (peroxisome proliferator activated receptor alpha), PDPK1 (3-phosphoinositide dependent protein kinase 1), HOXB7 (homeobox B7), IGF1 (insulin like growth factor 1), and SHBG (sex hormone binding globulin) (Figure 4). These are but a few examples of transcripts or proteins altered by PFNA.

Following gene set enrichment analysis for cell signaling pathways altered by PFNA as described above, the top 20 hits were revealed to be related to insulin signaling [“Insulin -> CEBPA/CTNNB/FOXA/FOXO”, “Insulin -> STAT Expression Targets”], immune system signaling [“TNF -> STAT Expression Targets”, “IL6 Expression Targets”, and “IL2 Expression Targets”], and growth hormone/prolactin signaling [“GH1/PRLR Expression Targets”, “PRL/GHR -> STAT Expression Targets”, “PRL/PRLR Expression Targets”] (Table 4). These regulated proteins and signaling pathways were linked to several diseases, including immunotoxicity, liver cancer, cardiotoxicity, and edema (Table 5). Many of these clinical signs/diseases have been observed previously in mammals and fish following PFNA exposure and other PFAS [88,98,99,100,101].

**Figure 5:**
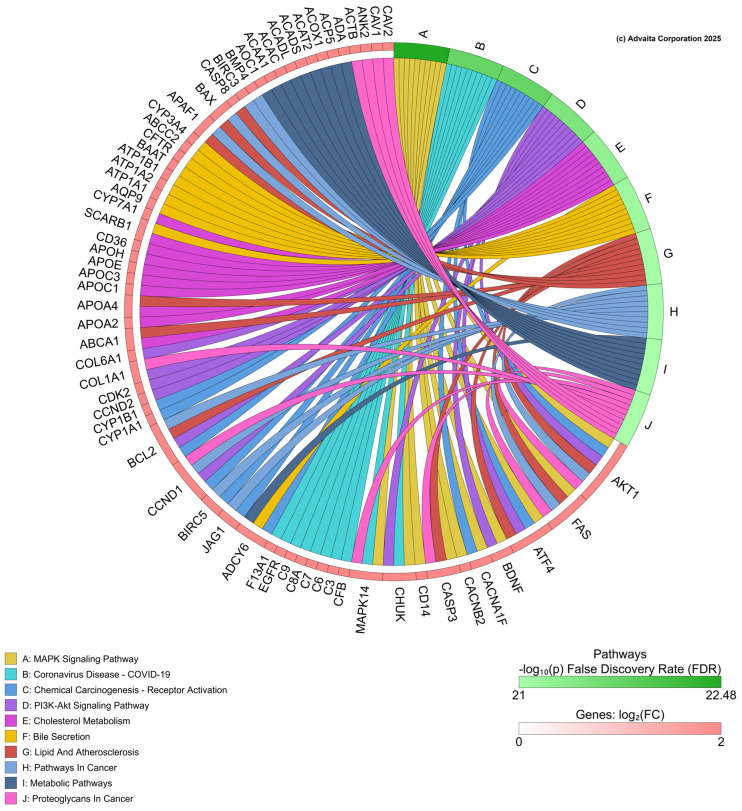
Chord diagram from iPathway shows the top biological pathways affected by perfluorononanoic acid (PFNA) exposure, based on transcript data collected from the Comparative Toxicogenomics Database.

For iPathway (https://apps.advaitabio.com/oauth-provider/ (accessed 20 January 2025)) analysis, all genes regulated by PFNA were imported from the CTD for PFNA. In this analysis, 2152 differentially expressed (DE) genes were imported and identified out of a total of 62,509 genes in the Advaita Knowledge Base (AKB). These data were analyzed in the context of pathways obtained from the Kyoto Encyclopedia of Genes and Genomes (KEGG) database (Release 110.0+/05-01, May 2024) [102,103], and gene ontologies from the Gene Ontology Consortium database. The iPathwayGuide scores pathways using the Impact Analysis method [104]. iPathway analyzed these data and generated a chord diagram of significant biological processes perturbed by PFNA based on the transcriptome data and gene list collected in CTD (Figure 5). The analysis revealed the following pathways as impacted by PFNA exposure, offering insight into putative targets for further study: MAPK signaling pathway, chemical carcinogenesis—receptor activation, PI3K-Akt signaling pathway and cholesterol metabolism among others. Pathways such as NF-kB pathway in zebrafish [105] and pathways related to metabolic processes like carbohydrate and lipid metabolism, DNA repair, thyroid hormones, and estrogen receptors have been shown to be affected by PFAS.

**Figure 6:**
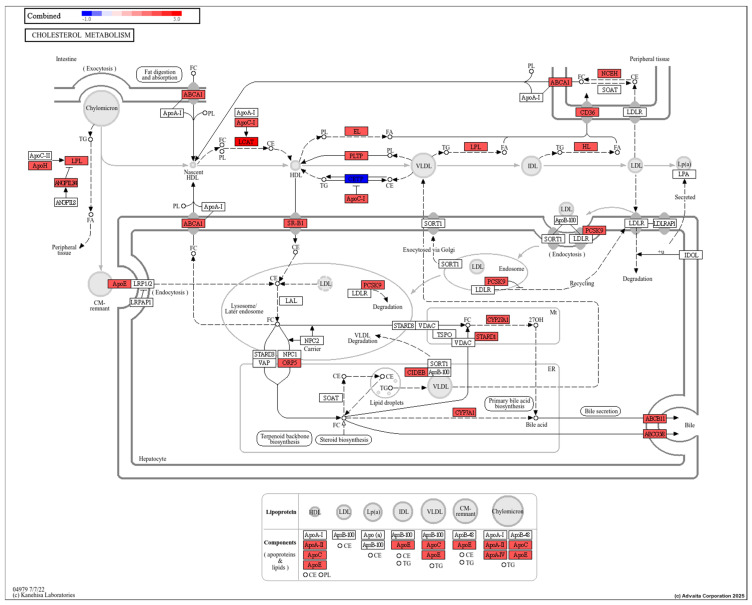
Cholesterol metabolism is negatively impacted by perfluorononanoic acid (PFNA) exposure. Red indicates the transcript has been identified in transcriptome or proteome studies to be altered by PFNA based on the Comparative Toxicogenomics Database. The red color does not indicate a particular change in expression, only whether its expression is altered by PFNA. The figure was generated by KEGG and utilized by iPathway. Blue color indicates a prediction for downregulation based on affected genes upstream.

Of note is cholesterol metabolism (Figure 6), which has altered transcripts in the pathway that include lipoprotein lipase, apolipoproteins, and enzymes (PCSK9, Cyp27A1 among others). Studies report similar outcomes of PFAS exposure on organisms. For example, Lin et al. [106] observed positive correlation between contents of the triglycerides and total cholesterol with PFAS accumulation in the liver of the black spotted frogs (*Pelophylax nigromaculatus*). Recently, Wang et al. [107] revealed that PFAS affects lipid metabolism through the activation of the PPAR pathway that promotes the transport of fatty acid and total cholesterol, β oxidation, and biosynthesis of fatty acids; subsequently accelerating fatty acid ester transformation from fatty acids and transformation to steroids from cholesterol. In another study, cholesterol metabolism and nuclear receptor pathways were enriched in Atlantic cod liver slices exposed to PFOS (10, 50, and 100 μM) for 48 h, and lipid biosynthesis was enriched after mixture exposures to PFNA, PFOA, and PFOS at equimolar concentrations [108]. Lastly, gene sets related to cholesterol biosynthesis have been repressed by PFAS (PFNA, PFOA, PFOS) in human HepaRG liver cells [109]. Taken together, there is strong evidence that cholesterol-related pathways are disturbed across different taxa; however, linking changes in gene expression to physiological changes requires determination of protein concentrations and activities.

## 7. Conclusions

In conclusion, while existing toxicity data for PFNA in fish is more extensive than for PFDA and PFUnDA, significant knowledge gaps persist among all three PFAS, including degradation pathways and kinetics, the influence of pH on their environmental behavior, and their occurrence in sludge and effluent. Based on our review, the toxicity of PFNA has been studied more often compared to PFDA and PFUnDA in fish. To date, no studies have measured the relationship between oxidative stress and PFUnDA exposure to fish. This is a research area that is lacking in information. Based on the gene ontology analysis to relate mRNA levels to pathway changes, prominent pathways affected by PFNA include insulin signaling [“Insulin -> CEBPA/CTNNB/FOXA/FOXO”, “Insulin -> STAT Expression Targets”] and growth hormone/prolactin signaling [“GH1/PRLR Expression Targets”, “PRL/GHR -> STAT Expression Targets”, “PRL/PRLR Expression Targets”]. This highlights the potential for far reaching effects on endocrine systems, and these impacts may not be limited to the thyroid and reproductive axes. Thus, toxicogenomics data indicate that growth and insulin signaling, as well as cancer-related pathways may be sensitive to perturbations by these longer-chained PFAS. Several transcripts related to cholesterol metabolism were also affected by PFNA. Additional research is required to evaluate their putative effects on lipid metabolism to more comprehensively evaluate the ecological risks associated with these compounds. It is unclear how these transcriptional alterations may translate into metabolic disorders, if at all. Overall, this review summarizes the current knowledge on the distribution, fate, and ecotoxicology of PFNA, PFDA, and PFUnDA in teleost fish, highlighting potential physiological and molecular responses that could aid in assessing long-chain PFAS toxicity in future studies.

## Figures and Tables

**Table 3:** List of transcripts perturbed by perfluorononanoic acid (PFNA), perfluorodecanoic acid (PFDA), and perfluoroundecanoic acid (PFUnDA) in zebrafish. These transcripts were manually compiled from literature.

PFAS	Process or Function	Upregulated Genes	Downregulated Genes	Reference
PFNA	Transport pathways	*slco2b1* at 14 dpf	*ap1s*, *slco2b1*, *tgfb1a* at 5 dpf	[35]
	Apoptosis	*jnk*, *aif*, *p53*	*pparαa*, *pparαb*, *bcl-2*	[87]
	Oxidative stress, lipid metabolism	*ucp2*, *lfabp*	*mt-nd1*, *sod1*, *cox1*, *mt-atp6*	Liu, Sheng [84]
	Thyroid disruption	*tpo*, *trα*, *ttr*, *ugt2a1*	*dio2*, *trβ*, *ugt1a5*	Liu, Wang [91]
	Reproduction	*erα*, *fshβ*, *cyp19b*, *lhβ*	*erβ*, *ar*, *fshr*, *lhr*, *star*, *fshr*, *3β-hsd*	[89]
	Cardiotoxicity	*amhc*, *nppa*, *nkx2.5*, *end1*, *tgfb2*		[93]
	Apoptosis, PPAR-signaling pathway	*angptl4*, *cyp24a1*, *elovl7b*, *hbbe3*, *hmgcra*, *hspb11*, *lyve1a*, *sqlea*, *ucp3*	*aqp8a.2*, *chia.1*, *cyp7a1*, *fabp10a*, *gck*, *mogat2*	[95]
PFDA	Endocrine disruption	*cyp19a*, *cyp19b*, *erα*, *er2β*, *fshβ*, *vtg1*		[90]
	Immune system apoptosis	*cxcl-c1c*, *il-8*, *il-β*, *tlr-4*, *tnf-α*		[86]
PFUnDA	Thyroid disruption		*trα*, *trβ*	[41]

**Table 4:** Top signaling pathways perturbed by perfluorononanoic acid (PFNA) as identified through transcript responses in animals using gene set enrichment analysis (Pathway Studio v12). The complete dataset is provided in the Supplementary Material. The table shows the total number of genes in the gene set as # of entities and number of expanded # of entities, percent overlap of the gene list (query) relative to all those in the pathway, and *p*-value based on the permutation test.

Name	# of Entities	Expanded # of Entities	Overlap	Percent Overlap	*p*-Value
TNF -> STAT Expression Targets	83	98	47	47	5.19 × 10^−21^
Insulin -> CEBPA/CTNNB/FOXA/FOXO Expression Targets	145	192	68	35	7.28 × 10^−21^
Insulin -> STAT Expression Targets	132	182	64	35	1.85 × 10^−19^
Insulin -> ELK/SRF/HIF1A/MYC/SREBF Expression Targets	138	208	64	30	4.28 × 10^−16^
PRL/GHR -> STAT Expression Targets	82	97	41	42	5.65 × 10^−16^
Insulin -> MEF/MYOD Expression Targets	148	199	62	31	6.72 × 10^−16^
IL6 Expression Targets	110	179	57	31	3.89 × 10^−15^
PRL/PRLR Expression Targets	78	93	39	41	4.21 × 10^−15^
IFNA1/IFNR Expression Targets	40	49	26	53	2.07 × 10^−13^
IL2 Expression Targets	97	138	46	33	3.48 × 10^−13^
GH1/GHR -> STAT Expression Targets	82	95	36	37	2.02 × 10^−12^
Leptin -> STAT Expression Targets	96	139	45	32	2.09 × 10^−12^
IFNG/IFNR Expression Targets	134	151	47	31	3.25 × 10^−12^
OSM/OSMR Expression Targets	37	55	26	47	6.76 × 10^−12^
TGFB1-ACVRL1 Expression Targets	221	306	73	23	6.95 × 10^−12^
EGF -> CTNN Expression Targets	143	167	49	29	1.25 × 10^−11^
CSF1 -> STAT Expression Targets	43	49	24	48	1.72 × 10^−11^
Cell Cycle Overiew	140	447	40	8	2.96 × 10^−11^
BMP4/BMPR2 Expression Targets	59	76	30	39	4.59 × 10^−11^
GH1/PRLR Expression Targets	58	69	28	40	9.61 × 10^−11^

**Table 5:** Diseases and clinical parameters associated with perfluorononanoic acid (PFNA) exposure (Pathway Studio v12, Elsevier).

Liver Injury	Dental Caries	Diabetes Mellitus	Hepatotoxicity
Hepatomegaly	Testicular toxicity	Death	Glucose intolerance
Atherosclerosis	Hyperbilirubinemia	Smoking	Liver cancer
Spermatogenic failure	Insulin resistance	Reproductive toxicity	Congenital malformation
Toxicity	Congenital heart defect	Hepatocellular carcinoma	Fatty liver
Metabolic syndrome X	Steatosis	Cholestasis	Immunotoxicity
Low birth weight	Body weight loss	Spinal deformity	
Developmental toxicity	Edema	Cardiotoxicity	

## Data Availability

Data will be made available upon request.

## Supplementary Materials

The following supporting information can be downloaded at: https://www.mdpi.com/article/10.3390/toxics13060436/s1, Table S1: Gene set enrichment for PFNA; Table S2: Abbreviations.
